# EXT1 methylation promotes proliferation and migration and predicts the clinical outcome of non‐small cell lung carcinoma via WNT signalling pathway

**DOI:** 10.1111/jcmm.16277

**Published:** 2021-02-09

**Authors:** Wencui Kong, Ying Chen, Zhongquan Zhao, Lei Zhang, Xiandong Lin, Xingguang Luo, Shuiliang Wang, Zhengbo Song, Xiangwu Lin, Guoxiang Lai, Zongyang Yu

**Affiliations:** ^1^ Department of Respiratory Medicine and Critical Care Medicine The 900th Hospital of Joint Logistic Support Force, PLA Fuzhou China; ^2^ Laboratory of Radiation Oncology and Radiobiology Fujian Cancer Hospital and Fujian Medical University Cancer Hospital Fuzhou China; ^3^ Division of Human Genetics Department of Psychiatry Yale University School of Medicine West Haven CT USA; ^4^ Department of Urology 900th Hospital of the Joint Logistics Team Support Force Fujian Medical University Fuzhou China; ^5^ Fujian Key Laboratory of Transplant Biology Affiliated Dongfang Hospital Xiamen University School of Medicine Fuzhou China; ^6^ Institute of Cancer and Basic Medicine Chinese Academy of Sciences Hangzhou China; ^7^ Department of Medical Oncology Cancer Hospital of the University of Chinese Academy of Sciences Hangzhou China; ^8^ Department of Medical Oncology Zhejiang Cancer Hospital Hangzhou China; ^9^ Medical Oncology The 900th Hospital of Joint Logistic Support Force, PLA Fuzhou China; ^10^ Department of Respiratory Medicine and Critical Care Medicine The 900th Hospital of Joint Logistic Support Force, PLA, Fujian Medical University，Affiliated Dongfang Hospital, Xiamen University School of Medicine, Fujian University of Traditional Chinese Medicine Fuzhou China

**Keywords:** DNA methylation, EXT1, migration, prognosis, proliferation, WNT

## Abstract

DNA methylation is important for lung cancer prognosis. In this work, it is aimed to seek novel biomarkers with DNA methylation‐expression‐pathway pattern and explore its underlying mechanism. Prognostic DNA methylation sites and mRNAs were screened in NSCLC data set from TCGA, and further validated using the samples retrospectively collected, and EXT1 was identified as a potential target. Gene body methylation of three CpG sites (cg03276982, cg11592677, cg16286281) on EXT1 was significantly associated with clinical outcome, and the EXT1 gene expression also predicted prognosis. The expression level of EXT1 was also correlated with its DNA methylation level. This observation was further validated in a new data set consist of 170 samples. Knocking down of EXT1 resulted in decreased proliferation and migration. EXT1 targets were analysed using GSEA. It is found that the WNT signalling is the potential downstream target of EXT1. Further analyses revealed that the EXT1 targets the beta‐catenin and effect migration rate of NSCLC cell lines. The WNT signalling inhibitor, XAV‐939, effectively disrupted the migration promotion effect induced by EXT1. In summary, EXT1 methylation regulates the gene expression, effects the proliferation and migration via WNT pathway and predicted a poor prognosis for NSCLC.

## 1 INTRODUCTION

Lung cancer is currently the leading cause of deaths in cancers. In China, 733 300 new cases and 610 200 deaths were estimated in 2015. Among the subtypes, non‐small cell lung carcinoma, which includes lung squamous cell carcinoma and adenocarcinoma, is the main subtype^1^ of lung cancer. The carcinogenesis and prognostic complexity of non‐small cell carcinoma were reflected by the genomic and environmental heterogeneity.^2^, ^3^

DNA methylation is the most well‐known epigenetic modification on mammal DNA, and the methylation pattern across genome significantly influence the DNA long‐term interaction, and chromatin structure and thus play vital roles in many biological processes, including carcinogenesis and cancer development. For example, the methylation status of APC and RARB was significantly altered during NSCLC carcinogenesis, and its methylation is associated the prognosis of NSCLC.^4^ Similarly, the promoter methylation of hOGG1 was shown to increase the risk of NSCLC carcinogenesis.^5^ Using the promoter methylation status of four genes (p16, CDH13, RASSF1A and APC), Brock et al developed a discrimination model for stage I lung cancer carcinogenesis and validated with external dataset.^6^ Additionally, LRP12 methylation was shown to predict the clinical response of NSCLC to carboplatin.^7^ However, none of these biomarkers were used clinically, due to the unknown mechanism of these genes.

The aim of this study was to screen the genes that effect the cancer development via DNA methylation alteration in non‐small cell lung carcinoma. Data in The Cancer Genome Atlas were selected and analysed, and EXT1 (exostosin glycosyltransferase 1) was identified as candidate gene. Subsequent analyses revealed that the DNA methylation of EXT1 regulates the expression level of EXT1 and thus promotes proliferation and migration. Further analyses revealed that WNT signalling pathway is the downstream of EXT1.

## 2 MATERIAL AND METHODS

### 2.1 Gene screening

The gene expression data of non‐small cell lung carcinoma in TCGA data set, which consist of lung squamous cell carcinoma and lung adenocarcinoma, were downloaded from UCSC Xena website (https://xena.ucsc.edu/). The primary tumour samples were enrolled whereas the other samples (metastatic and normal samples) were excluded. The gene expression values were transformed to log2(FPKM + 1) values from the normalized data. Cox univariate regression was implemented by correlating the gene expression levels and overall survival information, and *P* values were calculated (*P*1). In addition, the samples were divided into high‐expression and low‐expression groups according to the mRNA abundances. Subsequently, log rank test was carried out evaluate the survival difference between high/low‐expression group (*P*2). Genes with both *P*1 < 0.001 and *P*2 < 0.001 were identified as survival‐related genes (SRG).

DNA methylation data based on Illumina 450K array of NSCLC sample were also downloaded from UCSC Xena. The sample enrolment criteria were implemented as expression data. The raw data were normalized using quantile algorithm. Correlation between DNA methylation level and overall survival was analysed using both Cox univariate regression and log rank test, as expression data processing procedure, as survival‐related CpGs. Methylation sites significantly associated with survival using both mRNA (SRG) and methylation levels (SRC) were retained for further analyses.

The annotation information of the DNA methylation platform (Illumina 450K) was downloaded from the manufacture provided website. Genes annotated with at least 3 CpG sites were retained for further analysis.

### 2.2 RNA extraction, cDNA preparation and qRT‐PCR

Written consents have been achieved from the patients involved in this study. Total RNA from lung cancer cell lines (NCI‐H520 and A549, squamous cell carcinoma and adenocarcinoma) and the enrolled NSCLC samples was extracted using TRIzol (Invitrogen, CA) by following the manufacturer provided steps. The raw quality of the isolated RNA was evaluated by both Nanodrop 2000 and agarose gel electrophoresis. The cDNA was synthesized by templating 1‐2 μg total RNA with random primers and reverse transcriptase (Invitrogen, Los Angeles, CA, USA). Real‐time PCR was implemented using the SYBR Green kit (Applied TaKaRa, Tokyo, Japan) and ABI PRISM 7900 sequence detector. In this step, 18s RNA was used as the endogenous control, and the relative mRNA levels in different batches were calculated according to its relative Ct values and 18s RNA Ct values.

### 2.3 Western blot

Cells were cultured and retrieved in RIPA reagent. The cell lysate was centrifuged for 15 min at 15 000g, and the proteins were collected. After protein quantification by BSA Assay Kit, WB was implemented. The antibodies for GAPDH (Sigma; dilution at 1:5000, Shanghai, China), active β‐catenin (Merck Millipore, 05665; dilution at 1:3000, Shanghai, China) and total β‐catenin (BD Biosciences, 610154; dilution at 1:3000, Shanghai, China) were utilized. The signal intensity was detected and quantified by SuperSignalWest Femto system (Thermo Scientific, Shanghai, China).

### 2.4 Proliferation and migration assays

For migration assay, Transwell filter champers (Costar, Corning, NY, USA) were used according to the manufacturer provided manual. After culturing for 12 hours, six random microscopic fields were selected, and cell number in each field of each group was counted, and each experiment was repeated for 3 independent times. For the NSCLC cancer cell line proliferation assays, siEXT1 and control cells (4 × 10^3^ cells/well) were plated into 100 μL growth medium inside 96‐well plates for different time. Cell density of each period was evaluated by measuring cell density OD values with the Cell Counting Kit 8 assay (Dojindo Laboratories, Kumamoto, Japan) according to manufacturer provided manual.

### 2.5 DNA methylation quantification

A total of 160 NSCLC patients who underwent surgery at 900th Hospital of Joint Logistic Support Force Hospital, between December 2003 and March 2017 were enrolled in this study. All cases were histologically confirmed, and none of these patients received other therapy before surgery. Written informed consent were received from all patients involved according to the Guidelines of the Local Ethics Committee of 900th Hospital of Joint Logistic Support Force. DNA was extracted from tissues using a FFPE DNA Kit (Omega Bio‐Tek, Norcross, GA, USA) applying the manufacturer provided instructions. Extracted DNA concentrations were evaluated with a NanoDrop 2000 spectrophotometer (Thermo Fisher Scientific, Wilmington, DE, USA) and adjusted to approximately 40 ng/mL. Prepared DNA was subjected to bisulfite treatment using the EZ DNA Methylation‐Gold kit (ZYMO, Orange, CA, USA); converted DNA was dissolved in TE buffer and stored at or below −20°C for later use. Biotinylated reverse primers were substituted with 5′‐tailed unlabelled reverse primers, allowing single (expansive) biotinylated primers to be used for subsequent pyrosequencing™. PCR products (20 µL) were added to a mix comprising Streptavidin Sepharose HP™ (3 µL; GE Healthcare, Dornstadt, Germany) and binding buffer (37 µL; Qiagen, Hilden, Germany). The contents were mixed at 1400 rpm for 10 min at room temperature. Using the Vacuum Prep Tool™ (Qiagen), according to the manufacturer's instructions, single‐stranded PCR products were prepared. Sepharose beads with attached single‐stranded templates were released into a PSQ 96 Plate Low™ (Qiagen) containing a mix of 40 µL annealing buffer (Qiagen) and the corresponding sequencing primer at 400 nM. Pyrosequencing™ reactions were performed in a PyroMark ID System (Qiagen), according to the manufacturer's instructions, using the PyroMark Gold 96 Reagent Kit (Qiagen). CpG site quantification was performed using Pyro Q‐CpG™ methylation software (Qiagen).

### 2.6 Staining and treating with XAV‐939

H520 cells were seeded on a 6‐well plates and were treated with 2 μM lycopene for 6 hours; afterwards, the cells were fixed with cold methanol, treated with blocking 1% BSA, 0.1% gelatin buffer for 1 hour, incubated with β‐ catenin antibody for 1 hour, washed with PBS, treated using FITC‐conjugated mouse anti‐goat IgG antibody (Santa Cruz Biotechnology, Shanghai, China) for 1 hour, added 5 μg/mL of DAPI and place the cells to laser scanning confocal microscope analysis (Zeiss LSM510; Carl Zeiss AG Corporate, Shanghai, China) to generate β‐catenin was detected using confocal microscopy. XAV‐939 (Selleck, Huston, Texas) was diluted to 5 µM according to the manufacture provided manual and added to the cell lines to further diluted to 5 nM in both cell lines.

### 2.7 Statistical analyses

All analyses were implemented on R software and packages. Survival analyses were implemented on R package ‘survival’. Gene Set Enrichment analyses were carried out using java software ‘GSEA’^8^ released. Student's t test was used to assay clinical indicator comparison, the proliferation, DNA methylation and migration difference, and *P* < 0.05 was considered statistically significant.

## 3 RESULTS

### 3.1 Identification of genes on methylation‐expression‐prognosis axis for NSCLC

To systematically screen the genes in methylation‐expression‐prognosis way, the non‐small cell lung carcinoma samples, including lung adenocarcinoma and lung squamous cell carcinoma data set in The Cancer Genome Atlas (TCGA) data, were downloaded, combined and analysed. The DNA methylation difference between normal and NSCLC was identified, and CpG sites significantly associated with survival were also selected. In addition, gene expression profile was also analysed. Genes correlated with clinical outcome were also identified. Genes occurred simultaneously in both DNA methylation, and gene expression were retained. Furthermore, genes with multiple CpG sites inside its gene elements (TSS, promoter, gene body, UTR) were used for further analyses. The results were shown in Table 1. Genes with at least three CpG sites associated with clinical outcome of NSCLC were identified. The candidate genes were DKK1, EXT1, KIAA1324 and TRIM15. Among these genes, DKK1 has been widely reported for its prognostic value in NSCLC,^9^ and prognostic value of TRIM15 has been emphasized.^10^ Although the effect of KIAA1324 was also reported in NSCLC.^11^ Thus, the EXT1 DNA methylation and its function were studied in this work.

**TABLE 1:** The distribution of selected CpG sites and genes. These sites and genes listed were significantly associated with NSCLC survival

Genes	ID	Gene Elements	CpG_Island
DKK1	cg27411220	TSS1500	N_Shore
	cg07684796	1stExon	Island
	cg27591349	1stExon	Island
	cg11931116	5UTR	Island
	cg09445939	5UTR	Island
EXT1	cg03276982	Body	
	cg11592677	Body	
	cg16286281	Body	N_Shelf
KIAA1324	cg03919114	Body	S_Shore
	cg16797831	Body	S_Shore
	cg09820792	TSS200	Island
TRIM15	cg02012483	1stExon	
	cg01775921	Body	N_Shore
	cg12232118	5UTR	

### 3.2 Prognostic value of EXT1 in NSCLC

**FIGURE 1:**
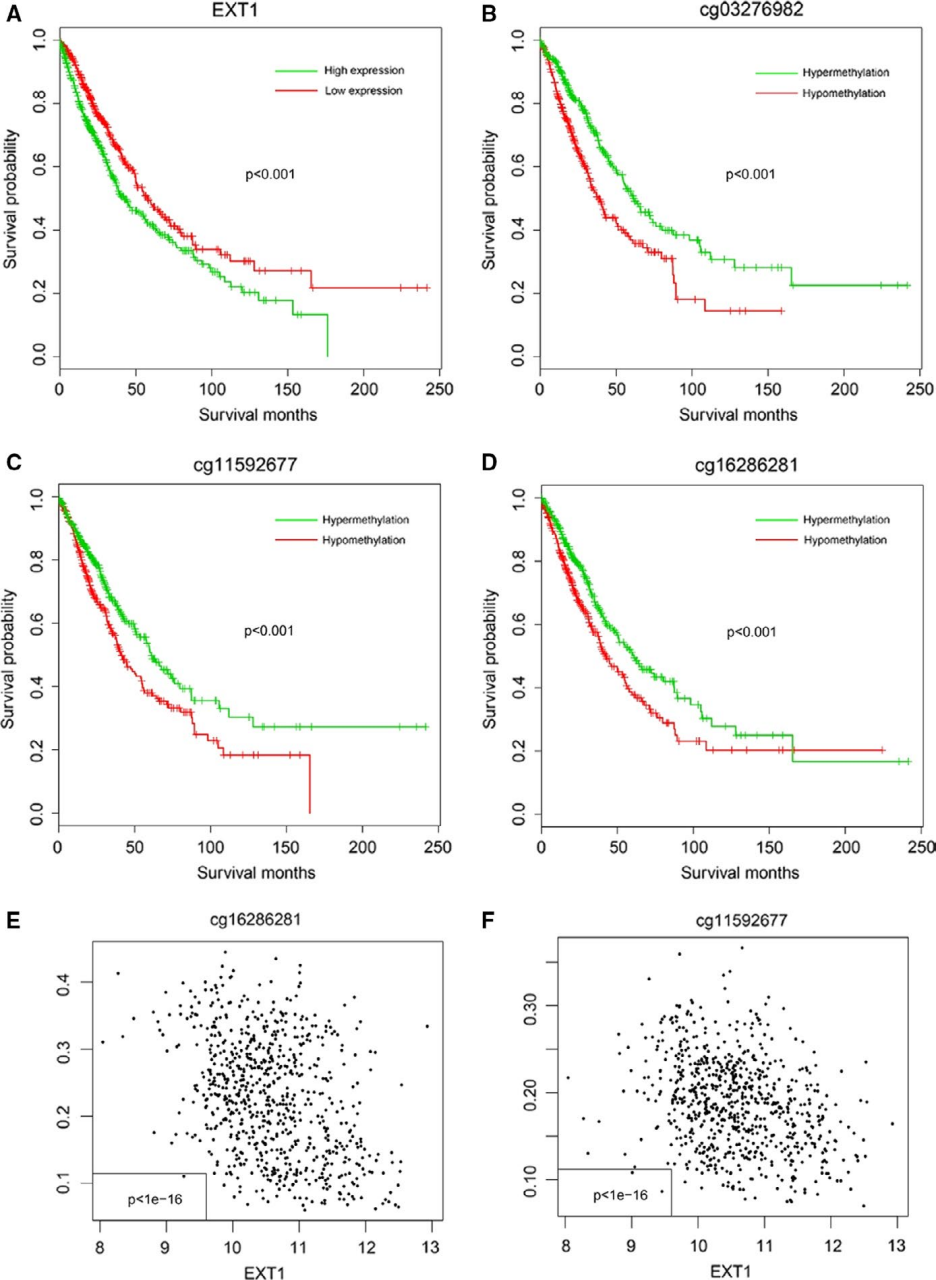
Prognostic value of EXT1 and cg03276982, cg11592677 and cg16286281 in TCGA cohort. The survival difference between high‐EXT1 and low‐EXT1 groups (A), hypermethylation and hypomethylation of cg03276982 (B), cg11592677 (C) and cg16286281 (D) was compared. The correlations between EXT1 mRNA and cg16286281 (E, left) and cg16286281 (F, right) were analysed

**FIGURE 2:**
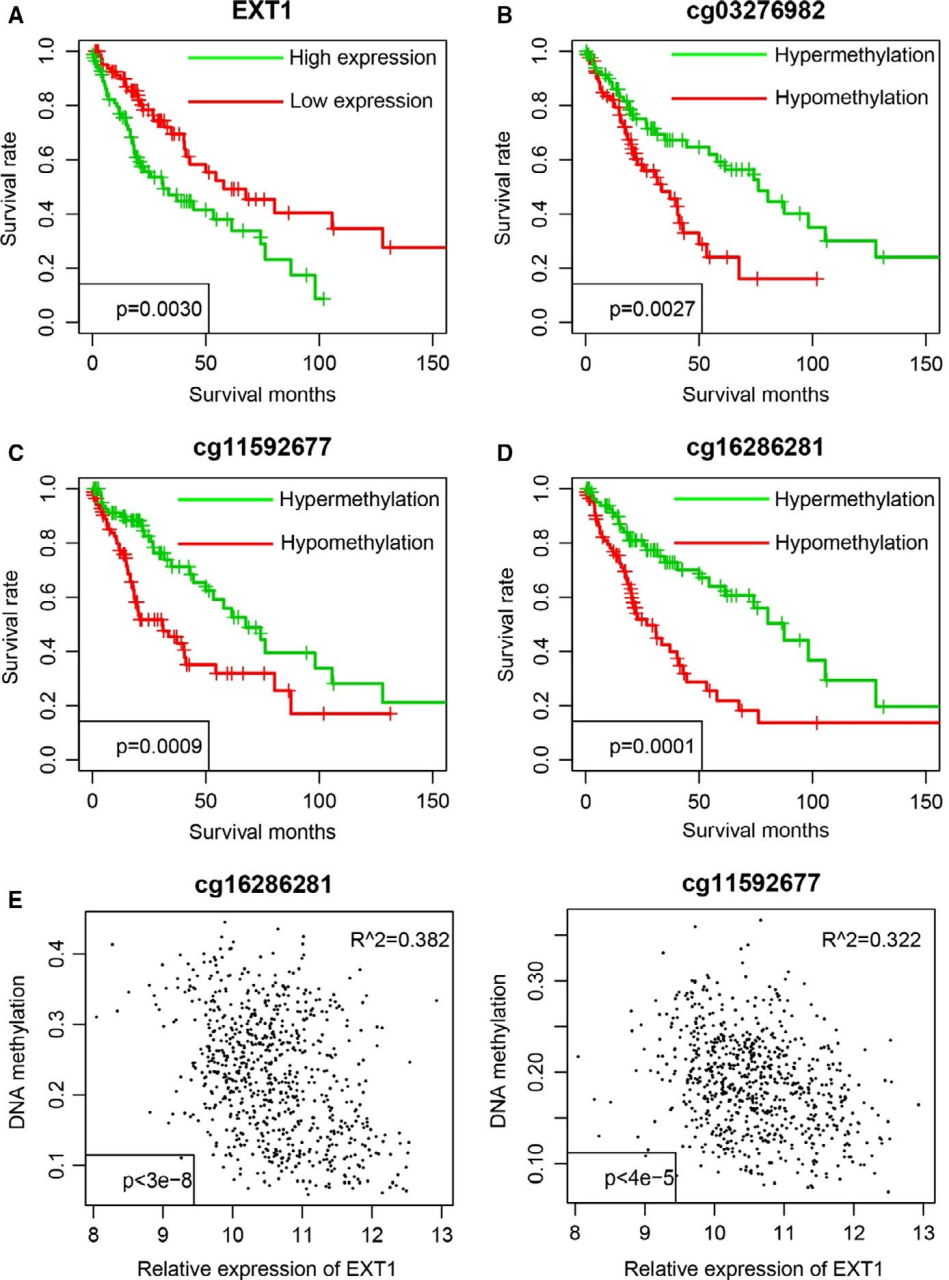
Prognostic values of EXT1 and cg03276982, cg11592677 and cg16286281 in our cohort. The survival difference between high‐EXT1 and low‐EXT1 group (A), hypermethylation and hypomethylation of cg03276982 (B), cg11592677 (C) and cg16286281 (D) were compared. The correlations between EXT1 mRNA and cg16286281 (E, left) and cg16286281 (E, right) were analysed

The prognostic value of EXT1 was analysed on both expression and DNA methylation levels. As expected, the NSCLC samples with high EXT1 expression were shown to significantly associated with worse prognosis (median survival months: 42.3, 95% CI: 36.9‐56.0), compared with the low‐expression group (median survival month: 57.9 95% CI: 57.9‐72.5, *P* = .00065), as shown in Figure 1A. The prognostic values of cg03276982, cg11592677 and cg16286281 methylation were also evaluated. As shown in Figure 1B‐D, the patients with hypomethylation of these sites had a significantly shorter survival period than the hypermethylated. The correlation between DNA methylation and EXT1 expression was also analysed. The mRNA abundance of EXT1 was negatively correlated with DNA methylation of cg11592677 and cg16286281 (Figure 2E,F, *P* < 1e‐16), but not cg03276982 (not shown). Collectively, these results indicate the DNA methylation level of EXT1 was negatively correlated with the expression of EXT1. In addition, the high mRNA expression and low DNA methylation of EXT1 suggested a worse survival in NSCLC patients.

### 3.3 Prognostic validation of EXT1 mRNA and methylation

Despite that the gene selection considered both mRNA expression and DNA methylation, the EXT1 selection was based on the data from TCGA cohort. Thus, the good performance of EXT1 for prognosis may resulted from over‐fitness. To exclude such reason, 170 newly primary NSCLC samples were collected from the 900th Hospital of Joint Logistic Support Force. Gene expression and DNA methylation levels of cg03276982, cg11592677 and cg16286281 were quantified using qRT‐PCR and Pyrosequencing methods, respectively. As expected, the expression of EXT1 was significantly associated overall survival of NSCLC (Figure 2A), and the similar DNA methylation pattern was observed in these three CpG sites (Figure 2B‐D). In consistent with previous results, the mRNA level of EXT1 was significantly and negatively associated with DNA methylation level of cg11592677 and cg16286281 (Figure 2E,F), but not cg03276982 (not shown). Collectively, these results indicate that the prognostic value of DNA methylation and mRNA abundance of EXT1 is robust across cohorts.

### 3.4 The Relationship between EXT1 and clinicopathological indicators

**FIGURE 3:**
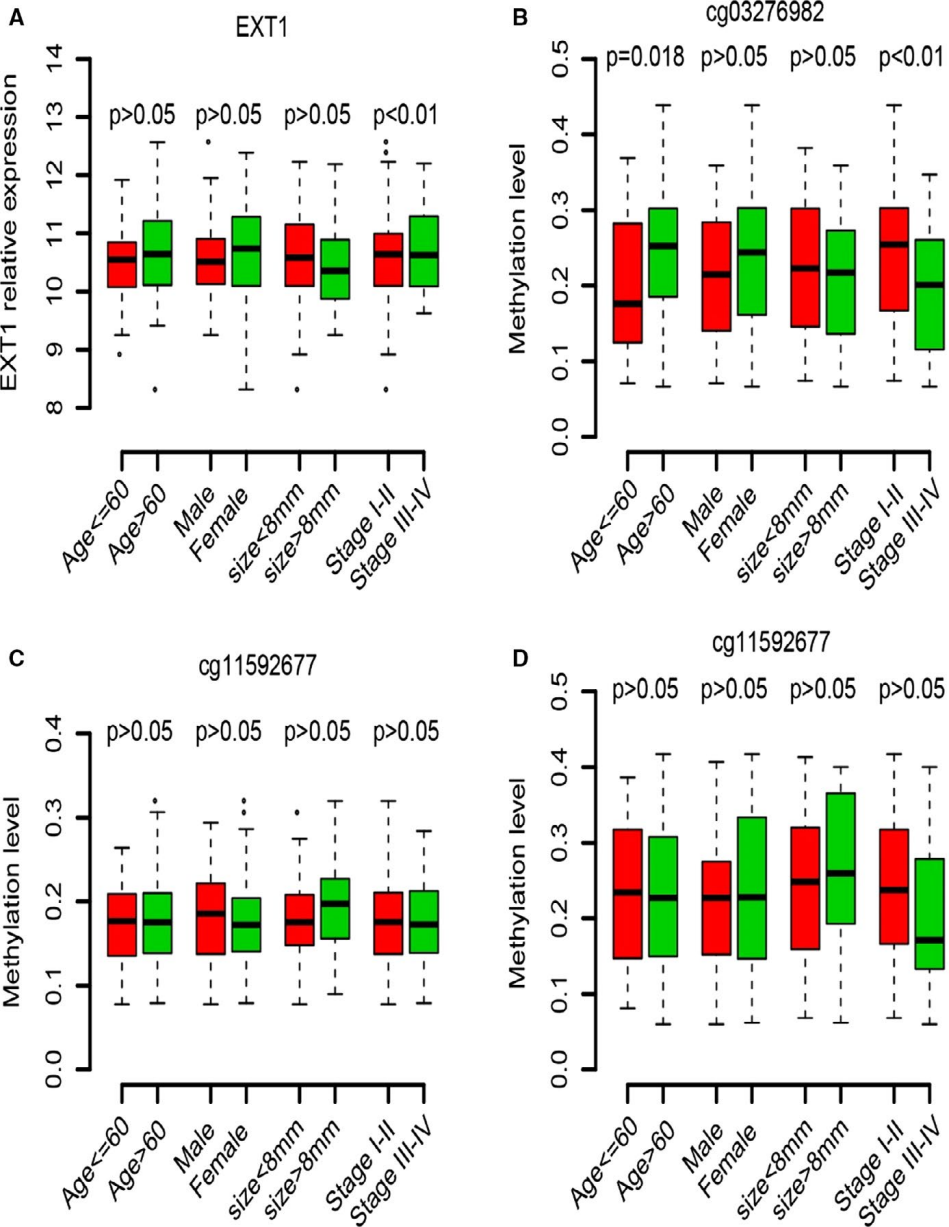
The correlation between EXT1 biomarkers and clinical indicators. The categorized clinical indicators are mostly independent from EXT1 expression (A), and methylation status of cg03276982 (B), cg11592677 (C) and cg16286281 (D)

The correlation between EXT1 and clinical indicators was also analysed in our own data set. Overall, the expression of EXT1 and methylation of CpG sites were independent from clinical indicators, with a few exceptions. As shown in Table 2, the expression of EXT1 was significantly associated with gender, and the methylation status of cg03276982 was correlated with age and primary tumour stage. In addition, relationship between clinical categories and EXT1 expression/methylation was also analysed. As shown in Figure 3, most clinical indicators are independent from EXT1 expression/methylation. Cox multivariate regression analysis was carried out to evaluate the importance of EXT1 and other clinical indicators. As shown in Table 3, EXT1 and all these CpG sites were significantly associated overall survival, whereas the other clinical indicators were not, indicating that EXT1 and its CpG sites are important indicator and powerful biomarker for the prognosis of NSCLC.

**TABLE 2:** The correlation between EXT1 biomarkers and clinical indicators. The bold categories are statistically significant (Fisher's exact test, *P* < .05)

	EXT1‐low	EXT1‐high	cg03276982‐low	cg03276982‐high	cg11592677‐low	cg11592677‐high	cg16286281‐low	cg16286281‐high
Age								
≤60	22	19	**28**	**13**	19	22	20	21
>60	61	63	**54**	**70**	63	61	62	62
Gender								
Female	**42**	**28**	40	30	31	39	35	35
Male	**43**	**57**	45	55	54	46	50	50
Size								
≤0.8	33	29	33	29	31	31	26	36
>0.8	19	13	17	15	12	20	12	20
Stage								
I‐II	64	66	**59**	**71**	64	66	61	69
III‐IV	21	19	**26**	**14**	21	19	24	16

**TABLE 3:** Significance of EXT1 biomarkers and clinical indicators. Multivariable regression between EXT1 biomarkers and the relevant *P* values

Comparison	Variables		*P* value	HR	ConfInt
EXT1	EXT1		0.003	1.812	1.2‐2.7
	Age		0.383	0.984	0.95‐1
	Gender	Male	Ref	/	/
		Female	0.144	1.607	0.85‐3
	Tumour Size		0.234	0.573	0.23‐1.4
	TNM Stage	StageI	Ref	/	/
		StageII	0.915	1.041	0.5‐2.2
		StageIII	0.248	1.590	0.72‐3.5
		StageIV	0.017	7.015	1.4‐35
cg03276982	cg03276982		0.017	0.005	0.00‐0.38
	Age		0.967	0.999	0.96‐1
	Gender	Male	Ref	/	/
		Female	0.144	1.607	0.85‐3
	iSize		0.235	0.568	0.22‐1.4
	Stage	StageI	Ref	/	/
		StageII	0.860	0.934	0.44‐2
		StageIII	0.481	1.343	0.59‐3.1
		StageIV	0.024	6.537	1.3‐33
cg11592677	cg11592677		0.000	0.000	0.00‐0.003
	Age		0.773	1.006	0.97‐1
	Gender	Male	Ref	/	/
		Female	0.144	1.607	0.85‐3
	iSize		0.488	0.723	0.29‐1.8
	Stage	StageI	Ref	/	/
		StageII	0.670	1.169	0.57‐2.4
		StageIII	0.210	1.639	0.76‐3.5
		StageIV	0.003	11.975	2.3‐62
cg16286281	cg16286281		0.000	0.000	0.00‐0.007
	Age		0.506	0.987	0.95‐1
	Gender	Male	Ref	/	/
		Female	0.144	1.607	0.85‐3
	iSize		0.406	0.672	0.26‐1.7
	Stage	StageI	Ref	/	/
		StageII	0.968	0.984	0.45‐2.1
		StageIII	0.873	1.071	0.46‐2.5
		StageIV	0.036	5.765	1.1‐30

### 3.5 The impact of oncogene EXT1 on cell proliferation and migration in NSCLC cell lines

**FIGURE 4:**
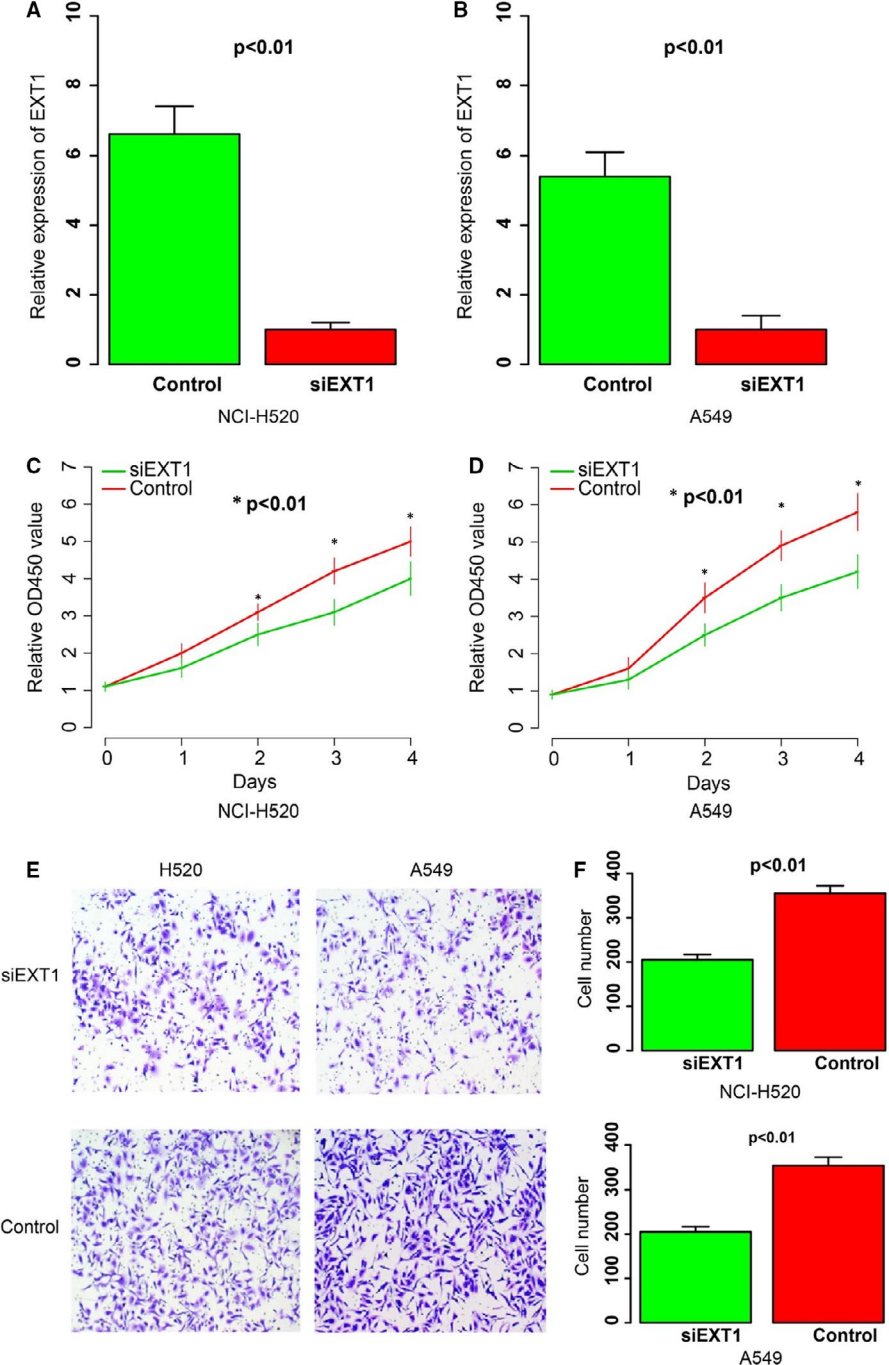
Impact of EXT1 on proliferation and migration. After knocking down of EXT1 with siRNA (A‐B), the proliferation rate in NCI‐H520 (C) and A549 (D) was significantly reduced, and the migration rate was also decreased by visualized by microscope (E) and counting (F)

To further investigate the role of EXT1 in NSCLC, two different NSCLC cell lines, NCI‐H520 and A549, were selected for functional validation. In this part, the impact of EXT1 on proliferation and migration was assayed. After knocking down of EXT1 in H520 and A549 (Figure 4A), the proliferation rate of both cell lines was determined and compared with the control group, using CCK9 assay in several days. As expected, the cell lines with EXT1 knocking down has a significantly lower proliferation rate than the control group (Figure 4B) from the 2nd day since culturing, indicating the role of EXT1 in cell cycle. Afterwards, the migration ability was also evaluated and compared between experimental and control group, and the migration rate of EXT1 knocking down group has a significantly less migration ability than the control (Figure 4C). All results above indicate that the EXT1 gene expression facilitates cell proliferation and migration in NSCLC cell lines.

### 3.6 Pathways associated with EXT1

**FIGURE 5:**
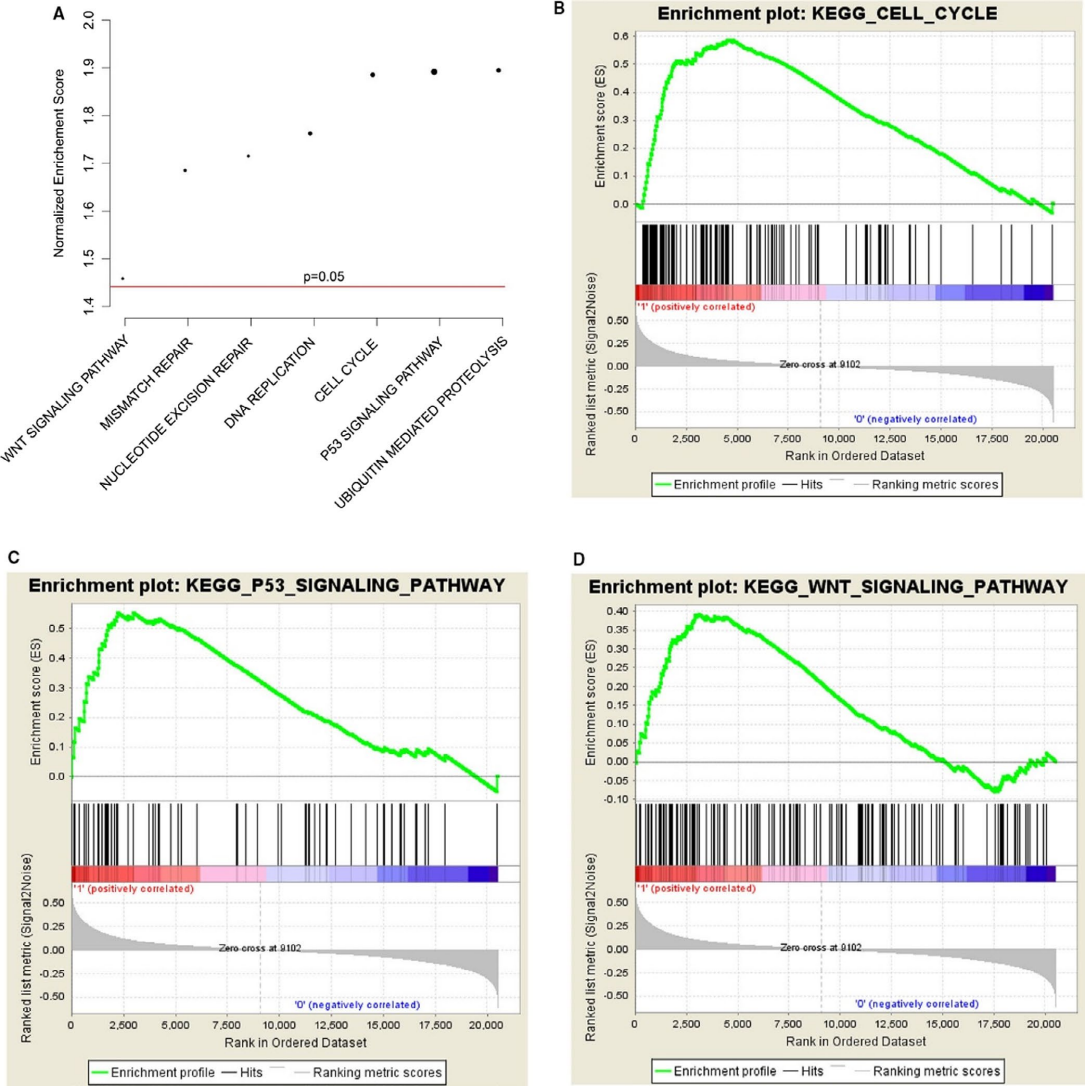
The potential targets of EXT1. Gene set enrichment analyses revealed that multiple pathways were altered (A), including cell cycle(B) P53 signaling (C) and WNT signalling (D)

To investigate the potential target genes downstream, Gene Set Enrichment Analysis (GSEA) was implemented by analysing EXT1‐high and EXT1‐low group, which were divided by median expression value, by using the data in TCGA data set. As shown in Figure 5A, cell cycle (Figure 5B), P53 signalling (Figure 5C), DNA repair and WNT signalling pathway (Figure 5D) were significantly enriched.

### 3.7 EXT1 effects the migration of NSCLC cell lines via WNT signalling pathway

**FIGURE 6:**
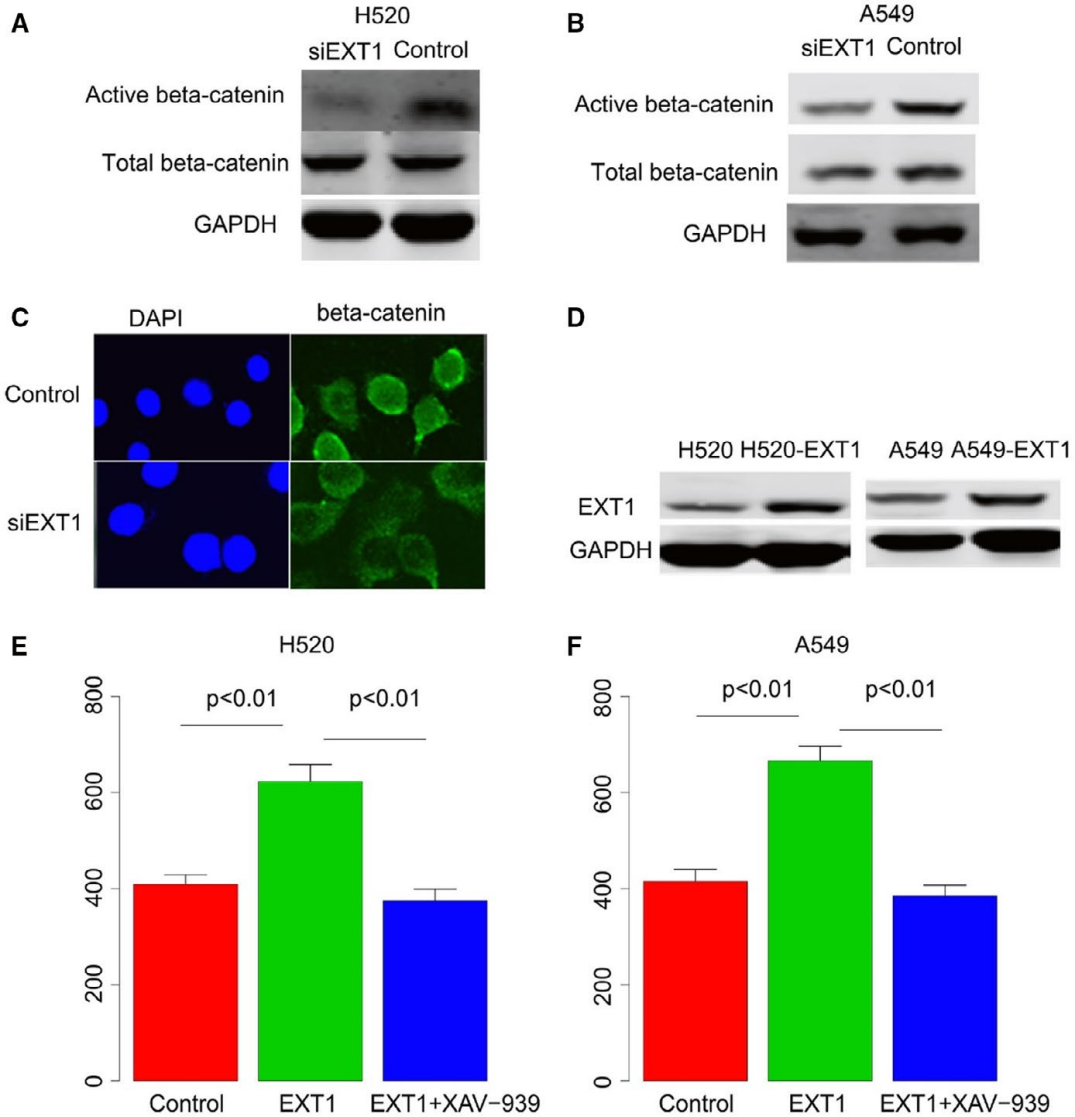
EXT1 effect migration via WNT signalling. Western blot validated that active beta‐catenin reduced when EXT1 was knocked down (A‐B), confocal microscope image revealed that the nucleus beta‐catenin decreased when EXT1 was knocked down (C). After overexpression of EXT1 in both cell lines (D), the migration rate increased (E‐F), whereas decreased after adding XAV‐939

As the impact of WNT signalling pathway has been emphasized in the other non‐cancer cell lines,^12^ we seek to investigate whether the EXT1 promotes cancer progression by WNT signalling pathway. After knocking down of EXT1 by siRNA, the active beta‐catenin protein abundance was compared with the control group. As expected, the beta‐catenin protein level was significantly decreased in both H520 and A549 cell lines (Figure 6A,B). For further validation, the beta‐catenin was visualized using confocal microscope. The beta‐catenin was observed, and its abundance is less in siEXT1 cell line (siEXT1‐H520) than the control (scramble‐H520), as shown in Figure 6C. To further validate whether the EXT1 promotes the migration via WNT signalling, the EXT1 overexpression H520 and A549 cell line were constructed (Figure 6D), and the migration rate was compared. Moreover, XAV‐939, a small molecular that inhibits the WNT signalling pathway, was added to the cells lines. The migration rate significantly reduced to appropriate control level (Figure 6E,F). Collectively, these results indicate that the EXT1 expression influence the WNT signalling pathway and thus promote the migration of NSCLC.

## 4 DISCUSSION

EXT1 (exostosin glycosyltransferase 1) is an endoplasmic reticulum‐resident type II transmembrane glycosyltransferase involved in the chain elongation step of heparan sulphate biosynthesis. It is ubiquitously expressed across tissues, in consistent with its promoter structure.^13^ This gene has been shown to be paly critical roles in lung development.^14^ This gene has been reported to have a frequent mutation in multiple osteochondromas,^15^, ^16^ and it is also a frequently mutated gene in lung cancer.^17^ In hepatocellular carcinoma, increased copy number of EXT1 results in more mRNA abundance and thus predicted a shorter disease‐free survival rate without vascular invasion.^18^ In breast cancer, EXT1 gene expression was shown to predict a risk of metastasis,^19^ and knocking down of EXT1 reduced the colony formation of breast cancer, and effect the therapy resistance.^20^ Moreover, it is reported that EXT1 promoter hypermethylation was detected in many types of cancers, including non‐melanoma skin cancer and leukaemia, especially acute promyelocytic leukaemia and acute lymphoblastic leukaemia.^21^

In previous report, EXT1 has been reported as to enhance canonical WNT signalling activity during chondrogenic differentiation,^12^ in addition, knock‐down of EXT1 using CRISPR/Cas9 resulted in aberrant WNT signalling in multiple myeloma.^22^ In this study, by comparing the transcriptome of NSCLC, we found that WNT signalling pathway was significantly different between EXT1‐high and EXT1‐low expression, and subsequent analyses also validated this observation. Functional assay revealed that the proliferation and migration ability of NSCLC cell lines. Although it is still unclear how EXT1 activates WNT signalling, study proposed that it modifies stroma interactions.^23^ Furthermore, the methylation status of EXT1 and the gene expression of EXT1 is significantly associated with overall survival of NSCLC. Both mRNA and DNA methylation performed better in survival prediction, compared with age, gender, pathological stages and primary tumour size.

Generally, DNA hypermethylation on promoter regions is correlated with depressed gene expression,^24^ whereas hypermethylation at gene body regions resulted in overexpression.^25^ cg03276982, cg11592677 and cg16286281 are all located in gene body and none of these sites located in CpG island. However, according to our results, the mRNA expression level of EXT1 was negatively correlated with methylation level of these sites, which is inconsistent with previous notion.

In addition to their anti‐inflammatory effect in smokers, omega‐3 polyunsaturated fatty acids (ω‐3 PUFAs) supplements are suggested to modulate pivotal pathways underlying the progression of lung cancer.^26^ As in the case of hepatocellular carcinoma, the latter may occur through inhibition of Wnt/β‐catenin signalling pathway.^27^ Thus, ω‐3 PUFAs act similarly to the WNT signalling inhibitor, XAV‐939 which effectively disrupts the migration promotion effect of EXT1. Consequently, administration of ω‐3 PUFAs may be potentially effective in counteracting the EXT1 methylation‐mediated lung cancer‐promoting effects. Collectively, this study revealed that gene body hypermethylation of EXT1 results in EXT1 overexpression, activates WNT signalling pathway and predicts the survival of NSCLC.

## CONFLICT OF INTEREST

The authors declare no (potential) conflict of interest.

## AUTHOR CONTRIBUTION

**Wencui Kong:** Conceptualization (equal); Data curation (equal); Formal analysis (equal); Methodology (equal); Software (equal); Writing‐original draft (equal); Writing‐review & editing (equal). **Ying Chen:** Conceptualization (equal); Data curation (equal); Investigation (equal); Methodology (equal); Visualization (equal); Writing‐original draft (equal); Writing‐review & editing (equal). **Zhongquan Zhao:** Conceptualization (equal); Data curation (equal); Supervision (equal); Writing‐original draft (equal); Writing‐review & editing (equal). **Lei Zhang:** Formal analysis (equal); Visualization (equal); Writing‐original draft (equal); Writing‐review & editing (equal). **Xiandong Lin:** Conceptualization (equal); Data curation (equal); Visualization (equal); Writing‐review & editing (equal). **Xingguang Luo:** Data curation (equal); Formal analysis (equal); Software (equal); Supervision (equal); Visualization (equal). **Shuiliang Wang:** Conceptualization (equal); Data curation (equal); Formal analysis (equal); Resources (equal); Software (equal). **Zhengbo Song:** Conceptualization (equal); Data curation (equal); Visualization (equal). **Xiangwu Lin:** Investigation (equal); Software (equal); Writing‐original draft (equal); Writing‐review & editing (equal). **Guo‐xiang Lai:** Conceptualization (equal); Data curation (equal); Formal analysis (equal); Writing‐original draft (equal); Writing‐review & editing (equal). **Zongyang Yu:** Conceptualization (equal); Data curation (equal); Formal analysis (equal); Investigation (equal); Project administration (equal); Software (equal); Supervision (equal); Visualization (equal); Writing‐original draft (equal); Writing‐review & editing (equal).

## ETHICS APPROVAL AND CONSENT TO PARTICIPATE

Written consents have been achieved from the patients involved in this study, and this study is approved by Ethnic Committee of The 900th Hospital of Joint Logistic Support Force.

## CONSENT FOR PUBLICATION

None.

## Data Availability

The data were all accessible via the accession provided in the material section.
